# Respiratory system toxicity induced by immune checkpoint inhibitors: A real-world study based on the FDA adverse event reporting system database

**DOI:** 10.3389/fonc.2022.941079

**Published:** 2022-08-19

**Authors:** Chanjuan Cui, Lei Deng, Wenqing Wang, Xiayang Ren, Yanfeng Wang, Wei Cui

**Affiliations:** ^1^ Department of Laboratory Medicine, National Cancer Center/National Clinical Research Center for Cancer/Cancer Hospital, Chinese Academy of Medical Sciences and Peking Union Medical College, Beijing, China; ^2^ Department of Radiation Oncology, National Cancer Center and Cancer Hospital, Chinese Academy of Medical Sciences and Peking Union Medical College, Beijing, China; ^3^ Department of Pharmacy, National Cancer Center and Cancer Hospital, Chinese Academy of Medical Sciences and Peking Union Medical College, Beijing, China; ^4^ Department of Comprehensive Oncology, National Cancer Center/National Clinical Research Center for Cancer/Cancer Hospital, Chinese Academy of Medical Sciences and Peking Union Medical College, Beijing, China

**Keywords:** immune checkpoint inhibitors, respiratory system, adverse events, faers, cancer

## Abstract

**Background:**

Immune checkpoint inhibitors (ICIs), the treatment of multiple cancer types, can be associated with respiratory system adverse events (AEs). The aim of this study is to quantify the association of respiratory system AEs and ICIs and to characterize the profiles of ICI-related respiratory system complications from Food and Drug Administration Adverse Event Reporting System (FAERS) data.

**Methods:**

The disproportionality of respiratory system AE-related ICIs based on FAERS data from January 2014 to September 2021 was analyzed using the reporting odds ratio (ROR) and information component (IC) as measures of potential risk increase.

**Results:**

A total of 38,415,849 records were involved; among these, 36,923 records related to respiratory system AEs after ICI treatment were identified. In the first 3 months, the cumulative proportion of respiratory system AEs was 75.40%. Men had a slightly higher reporting frequency than that of women (ROR = 1.74, 95% CI: 1.70–1.78). Death cases had a slightly higher reporting frequency in ICI-associated respiratory system AEs than that of other drug-associated respiratory system AEs (ROR = 1.40, 95% CI: 1.38–1.41). Anti-programmed cell death 1 (PD-1) drugs and anti-programmed cell death ligand 1 (PD-L1) drugs were significantly associated with respiratory system toxicities. However, anti-cytotoxic T lymphocyte-associated protein 4 (CTLA-4) drugs did not demonstrate an association with respiratory system toxicities. Interstitial lung disease and pneumonitis were found to be significantly associated with all eight types of ICIs. In addition, 7 in 10 class-specific respiratory system AEs (lower respiratory tract disorders, pleural disorders, pulmonary vascular disorders, respiratory disorders not elsewhere classified (NEC), respiratory tract infections, respiratory tract neoplasms, and thoracic disorders) were associated with ICIs. The signal values of IC_025_ were from 0.08 to 2.66.

**Conclusions:**

Overall, this study showed a high reporting frequency of respiratory system toxicities caused by ICIs. Early recognition and management of ICI-related respiratory system AEs are of vital importance in practice. Maximizing the benefit while reducing potential respiratory system toxicities of ICIs should become a priority.

## Introduction

Immune checkpoint inhibitors (ICIs) have become prevalent in the treatment of multiple cancer types (1–7). The recently approved ICIs include anti-cytotoxic T lymphocyte-associated protein 4 (CTLA-4), anti-programmed cell death 1 (PD-1), and anti-programmed cell death ligand 1 (PD-L1) (8, 9). By removing the inhibitory effect and releasing the restraints on the antitumor immune response (10), ICIs have shown significant efficacy and improved clinical outcomes during the treatment of a variety of solid tumours, such as lung cancer, melanoma, renal cell carcinoma, and urothelial carcinoma (3, 4, 11). However, with the increasing use of ICIs in practice, immune-related adverse events (AEs) are increasingly being appreciated (12–14). Respiratory system immune-related AEs are one of the most common (15–17). Cases of respiratory system AEs have been pointed out since the first clinical trials on ICIs. Moreover, immune-mediated pneumonia was one of the most common respiratory system immune-related AEs. But other respiratory system AEs have also been reported recently, such as diaphragm myositis and sarcoid-like granulomas (18, 19). However, due to relatively small sample sizes and limited follow-up time, it was difficult to evaluate sequelae of characteristics of respiratory system toxicities from ICIs. Notably, some respiratory system immune-related AEs could cause serious outcomes in practice. Given the increasing number of patients with cancer expected to be treated with ICIs in the coming years, more attention needs to be paid to these respiratory toxicity issues.

The Food and Drug Administration Adverse Event Reporting System (FAERS) is one of the largest pharmacovigilance databases on AE reports from real-world data (19–21). By mining large samples from the FAERS, it could be possible to better obtain clinical characterization of AEs, such as onset time, outcomes, and prognosis (20). In this study, we aimed to conduct a disproportionality analysis leveraging FAERS to systematically characterize and assess ICI monotherapy-associated respiratory system toxicities.

## Materials and methods

### Study design and data sources

This observational pharmacovigilance study is a disproportionality analysis based on the FAERS database covering the period from January 2014 to September 2021. The FAERS database is a large postmarketing database for the safety surveillance of a drug. Currently, millions of AE reports are submitted to this database by health care professionals, consumers, manufacturers, etc. The FAERS files updates every quarter online. All these data are available at https://fis.fda.gov/extensions/FPD-QDE-FAERS/FPD-QDE-FAERS.html.

Variables such as Case Identification (CASEID), age, sex, event date, drug names, and outcomes were extracted in each report. Moreover, we removed duplicated records using the FDA’s recommended method by choosing the latest FDA_DT when the CASEID was the same and selecting the higher PRIMARYID when the CASEID and FDA_DT were the same. In the FAERS database, AEs are coded by the preferred term (PT) according to the Medical Dictionary for Regulatory Activities (MedDRA) (Version 24.1 English). A specific PT can be assigned to several high-level terms (HLTs), high-level group terms (HLGTs), and system organ classes (SOCs). In this analysis, we categorized respiratory system entities according to 10 categories of SOCs (bronchial disorders, lower respiratory tract disorders, pleural disorders, pulmonary vascular disorders, respiratory disorders NEC, respiratory tract infections, respiratory tract neoplasms, respiratory tract signs and symptoms, thoracic disorders, and upper respiratory tract disorders) (**Table 1**). More details of the SOCs used in our study can be accessed in the **Supplementary Materials** (**Table S1**).

**Table 1:** Respiratory system event groups according to MedDRA 24.1.

MedDRA Term	MedDRA Code
Bronchial disorders	10006436
Lower respiratory tract disorders	10024967
Pleural disorders	10035597
Pulmonary vascular disorders	10037454
Respiratory disorders NEC	10038716
Respiratory tract infections	10024970
Respiratory tract neoplasms	10029107
Respiratory tract signs and symptoms	10079101
Thoracic disorders	10013369
Upper respiratory tract disorders	10046304

ICIs in this study include anti-PD-1 antibodies (nivolumab, cemiplimab, and pembrolizumab), anti-PD-L1 antibodies (atezolizumab, avelumab, and durvalumab), and anti-CTLA-4 antibodies (ipilimumab and tremelimumab). Details for these drug names are listed in the **Supplementary Material** (**Table S2**).

### Statistical analysis

In this study, disproportionality analysis was applied to evaluate whether suspected target respiratory system AEs were differentially reported between ICIs and other drugs in the FAERS database. The degree of disproportionality was calculated through the reporting odds ratio (ROR) and Bayesian confidence propagation neural networks of information components (ICs). The criteria of a significant signal were identified by the 95% confidence interval lower end for both ROR (ROR_025_) and IC (IC_025_). If ROR_025_ was higher than one or IC_025_ exceeded zero, it was considered statistically significant to detect a potential signal. A statistical shrinkage transformation model was applied to obtain robust results (11). In order to reduce false-negative adverse signals, the statistical shrinkage transformation was originally recommended by the World Health Organization Uppsala Monitoring Center. The formula is as follows:


$$\begin{array}{l} N_{Expected}=(N_{Drug}*N_{Event})/NTotal \end{array}$$

$$ROR=(N_{Observrd}+0.5)/(N_{Expected}+0.5)$$

$$IC=\log2[(N_{Observrd}+0.5/(N_{Expected}+0.5)]$$

N_Expected_ is the number of records expected for the target drug AE combination;
  N_Observed_ is the number of observed target drug AE records;
  N_Drug_ is the number of any target drug-associated AE records;
  N_Event_ is the number of target AE records;
  N_Total_ is the total number of any AE records for any drug.
  The time to onset of AEs was carried out according to the formula as follows: Time to onset = Event date – Therapy start date.

## Results

### Data selection

**Figure 1:**
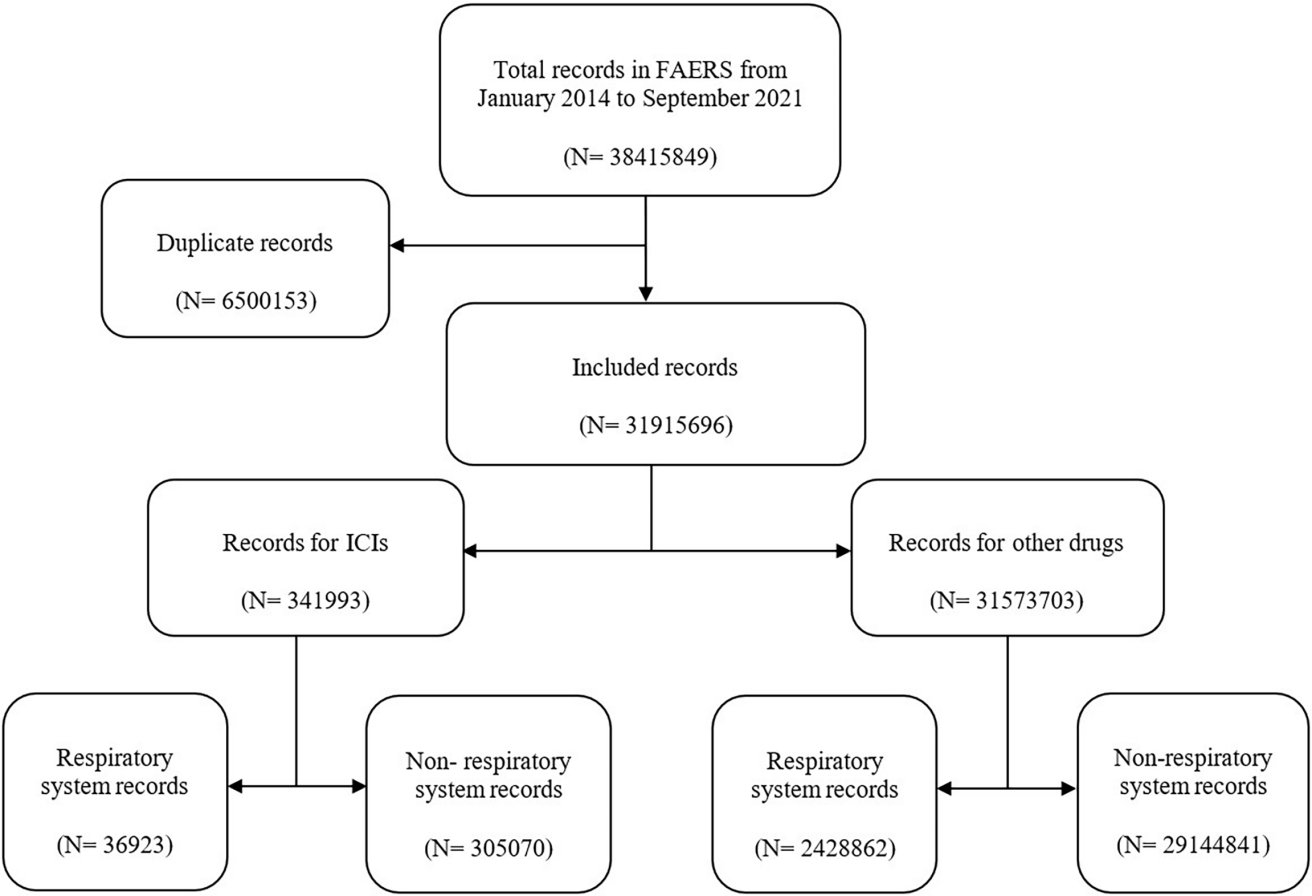
The selection process of adverse event records. ICIs, immune checkpoint inhibitors.

In this study, a total of 38,415,849 records were extracted from the FAERS database (**Figure 1**). After exclusion of duplicates, the number of records was 31,915,696, among which 341,993 reports were associated with ICI-related AEs. Then, 36,923 records were found to be associated with respiratory system AEs in ICI-related AEs. Moreover, 2,428,862 records were related to respiratory system AEs in other drug AEs.

### Baseline characteristics

The baseline respiratory system AEs for the ICI and control groups are listed in **Table 2**. Most cases were reported in 2017–2021, suggesting the substantially increased use of ICIs in recent years. The ICI-related respiratory system AE records were mainly from the United States (11,930, 32.31%), Japan (7,911, 21.43%), France (3,234, 8.76%), and Germany (2,526, 6.84%). Regarding respiratory system AEs, men accounted for a larger proportion than that of women. A total of 22,532 were reported in (61.02%) male patients, while 11,734 (31.78%) were reported in female patients. After further disproportionality analysis, the results showed that men had a slightly higher reporting frequency than that of women (ROR = 1.74, 95% CI: 1.70–1.78). Moreover, 11,314 (30.64%) death cases were related to ICI-associated respiratory system AEs at levels higher than those of respiratory system AEs related to other drugs (74,758, 24.51%). Upon further analysis, death cases had a slightly higher reporting frequency in ICI-associated respiratory system AEs than that of other drug-associated respiratory system AEs (ROR = 1.40, 95% CI: 1.38–1.41). In addition, patients aged ≥ 70 years accounted for a lower proportion of respiratory system AEs than those aged<70 years (30.83% vs. 50.22%). But disproportionality analysis showed that the two age groups were not significantly different (ROR = 0.86, 95% CI: 0.84–0.88).

**Table 2:** Characteristics of respiratory system AEs in the ICI group and control group.

Characteristics	Respiratory System AEs in ICIs (n = 36,923)	Respiratory System AEs in other drugs (n = 2,428,862)
Gender		
Men	22,532 (61.02%)	841,590 (34.65%)
Women	11,734 (31.78%)	1,413,861 (58.21%)
Missing	2,657 (7.20%)	173,411 (7.14%)
Age		
≥70 years	11,383 (30.83%)	467,185 (19.23%)
<70 years	18,543 (50.22%)	1,224,142 (50.40%)
Missing	6,997 (18.95%)	737,535 (30.37%)
Year		
2014	318 (0.86%)	298,607 (12.9%)
2015	1,001(2.71%)	304,941 (12.55%)
2016	2,202 (5.96%)	248,505 (10.23%)
2017	4,395 (11.90%)	259,190 (10.67%)
2018	6,482 (17.56%)	306,827 (12.63%)
2019	7,175 (19.43%)	302,709 (12.46%)
2020	7,167 (19.41%)	347,712 (14.32%)
2021	8,183 (22.16%)	360,371 (14.84%)
Outcome		
Death	11,314 (30.64%)	264,633 (10.90%)
Life-threatening	2,322 (6.29%)	107,634 (4.43%)
Disability	388 (1.05%)	41,057 (1.69%)
Hospitalization	13,622 (36.89%)	728,895 (30.01%)
Congenital anomaly	3 (0.01%)	7,930 (0.33%)
Other serious	7,589 (20.55%)	649,199 (26.73%)
Required intervention	0 (0.00%)	0 (0.00%)
Missing	1,685( 4.56%)	629,514 (25.92%)
Reporting country		
United States	11,930 (32.31%)	1,408,884 (58.01%)
Japan	7,911(21.43%)	74,822 (3.08%)
France	3,234 (8.76%)	67,767 (2.79%)
Germany	2,526 (6.84%)	68,594 (2.82%)
Italy	982 (2.66%)	34,626 (1.43%)
Great Britain	1,250 (3.39%)	100,978 (4.16%)
Canada	1,567 (4.24%)	263,851 (10.86%)
Spain	587 (1.59%)	21,221 (0.87%)
Australia	528 (1.43%)	19,664 (0.81%)
Netherlands	230 (0.62%)	14,061 (0.58%)
Others	6,045 (16.37%)	277,477 (11.42%)
Missing	133 (0.36%)	76,917 (3.17%)

AEs, adverse events; ICIs, immune checkpoint inhibitors.

### Time to onset

**Figure 2:**
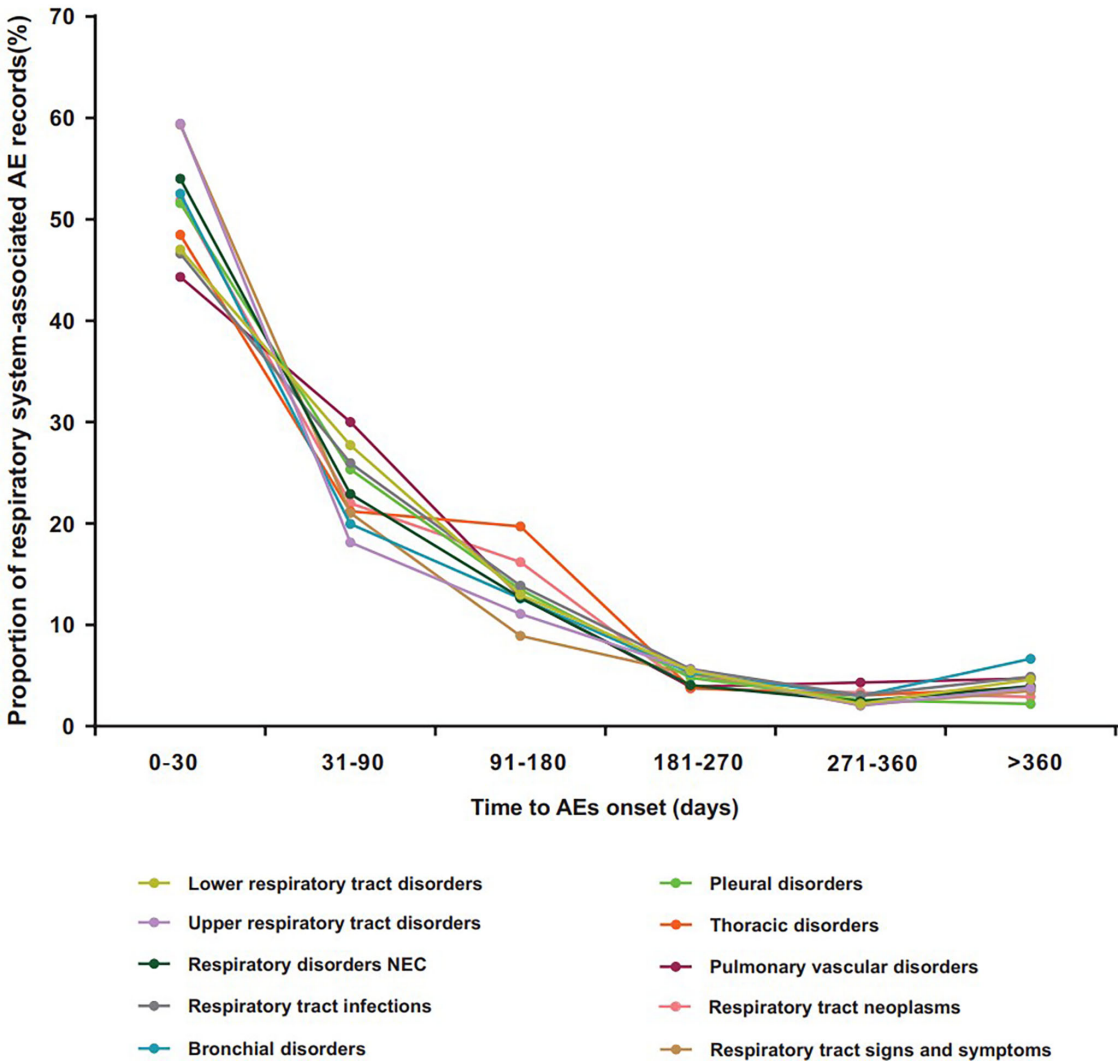
Time to onset for ICI-related class-specific respiratory system AEs. AEs, adverse events; ICIs, immune checkpoint inhibitors.

**Figure 2** shows the differential spectra of time to onset in ICI-related class-specific respiratory system AEs. After exclusion of records without event time, a total of 17,999 records covered the onset time of ICI-related respiratory system AEs. Overall, the median onset time of respiratory system AEs was 36 days (Q1–Q3: 14–98 days) after ICI initiation for all categories (**Table S4**). The cumulative proportion of respiratory system AE records that occurred at the 1-month landmark (50.58%, 9,014) was higher than that at other times (**Figure 2**, **Table S3**). Within 3 months, the cumulative proportion of respiratory system AE records was 75.40% (13,571) (**Figure 2**, **Table S3**). Data on upper respiratory tract disorders showed the shortest median time of 28 days. Pulmonary vascular disorders and thoracic disorders had the longest median time of 42 days (**Table S4**).

### Outcome

**Figure 3:**
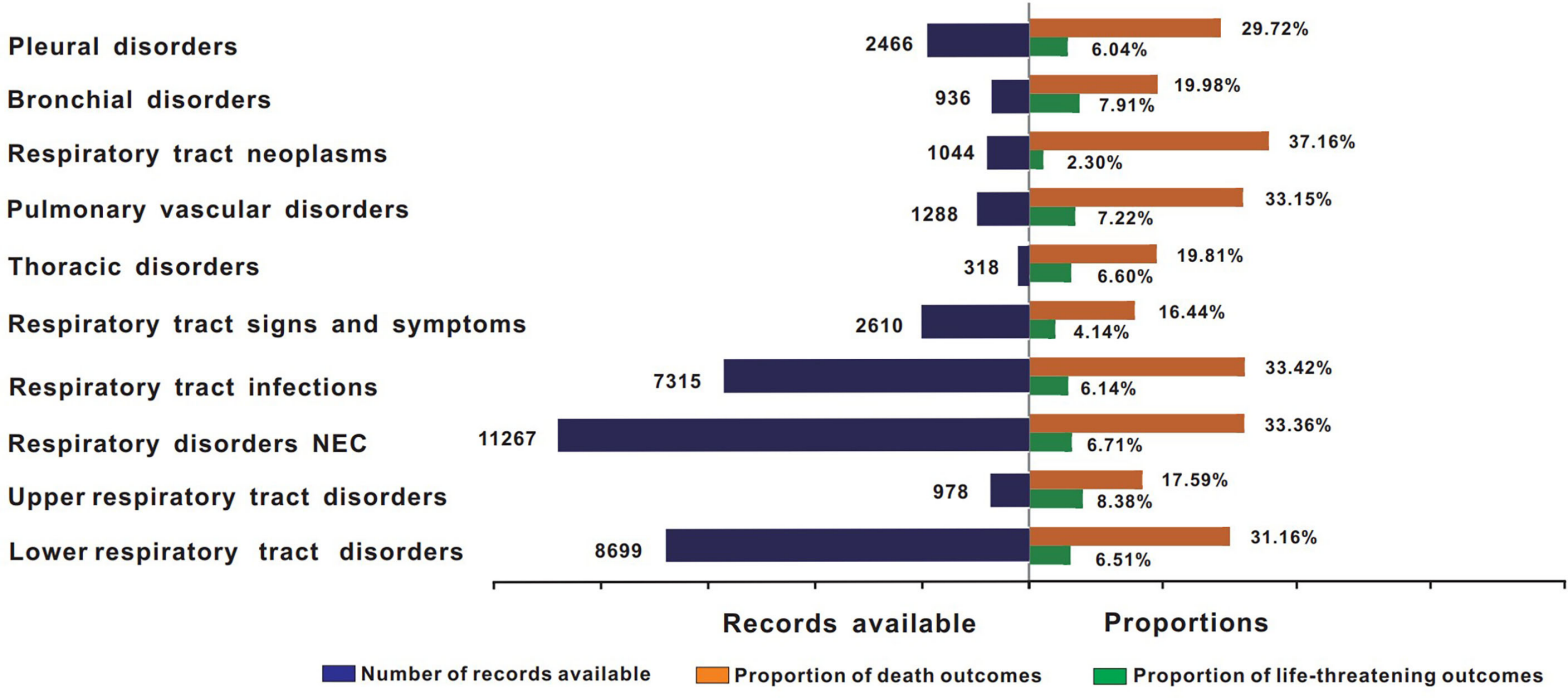
Records and proportions of death in class-specific respiratory system AEs. AEs, adverse events; ICIs, immune checkpoint inhibitors.

**Figure 3** shows the death and life-threatening proportions according to the types of respiratory system AEs. In general, death accounted for 30.64% of all ICI-associated respiratory system AE records with available final outcome information (**Table 2**). Further analysis showed that the severity of these events varied. In total, lower respiratory tract disorders, respiratory disorders NEC, respiratory tract infections, respiratory tract neoplasms, pulmonary vascular disorders, and pleural disorders were the six conditions with the highest proportions of death in all ICI regimens (31.16%, 33.36%, 33.42%, 37.16%, 33.15%, and 29.72%, respectively).

### Disproportionality analysis

The signal values and the association between class-specific respiratory system AEs and ICIs are shown in **Table 3**. Seven in 10 class-specific respiratory system AEs (lower respiratory tract disorders, pleural disorders, pulmonary vascular disorders, respiratory disorders NEC, respiratory tract infections, respiratory tract neoplasms, and thoracic disorders) were associated with ICIs. The signal values of IC_025_ were from 0.08 to 2.66. Among these AEs, respiratory disorders NEC (N = 11,267, 30.52%), lower respiratory tract disorders (N = 8,699, 23.56%), and respiratory tract infections (N = 7,315, 19.81%) largely comprised the reported problems. Notably, the magnitude of the disproportionality association was the highest for lower respiratory tract disorders (IC_025_ = 2.66, ROR_025_ = 6.33).

**Table 3:** Results of the disproportionality analysis for class-specific respiratory system AEs associated with ICIs.

Respiratory system events	N	IC	IC_025_	IC_975_	ROR	ROR_025_	ROR_975_
Bronchial disorders	936	-0.75	-0.87	-0.67	0.59	0.56	0.63
Lower respiratory tract disorders	8,699	2.69	**2.66**	2.72	6.47	**6.33**	6.61
Pleural disorders	2,466	2.18	**2.15**	2.23	4.54	**4.36**	4.73
Pulmonary vascular disorders	1,288	0.59	**0.50**	0.66	1.50	**1.42**	1.59
Respiratory disorders NEC	11,267	0.48	**0.44**	0.50	1.39	**1.36**	1.42
Respiratory tract infections	7,315	0.12	**0.08**	0.15	1.08	**1.06**	1.11
Respiratory tract neoplasms	1,044	0.96	**0.86**	1.04	1.95	**1.83**	2.07
Respiratory tract signs and symptoms	2,610	-0.77	-0.83	-0.72	0.59	0.56	0.61
Thoracic disorders	318	0.48	**0.30**	0.62	1.40	**1.25**	1.56
Upper respiratory tract disorders	978	-1.00	-1.11	-0.95	0.50	0.47	0.53

AEs, adverse events; ICIs, immune checkpoint inhibitors; N, number of records; IC_025_, the lower limit of a 95% CI for the IC; IC_975_, the upper limit of a 95% CI; ROR_025_, the lower limit of the 95% CI of ROR; ROR_975_, the upper limit of the 95% CI of ROR. IC_025_ >0 and ROR_025_ >1 were deemed a signal (bold mark).

**Figure 4:**
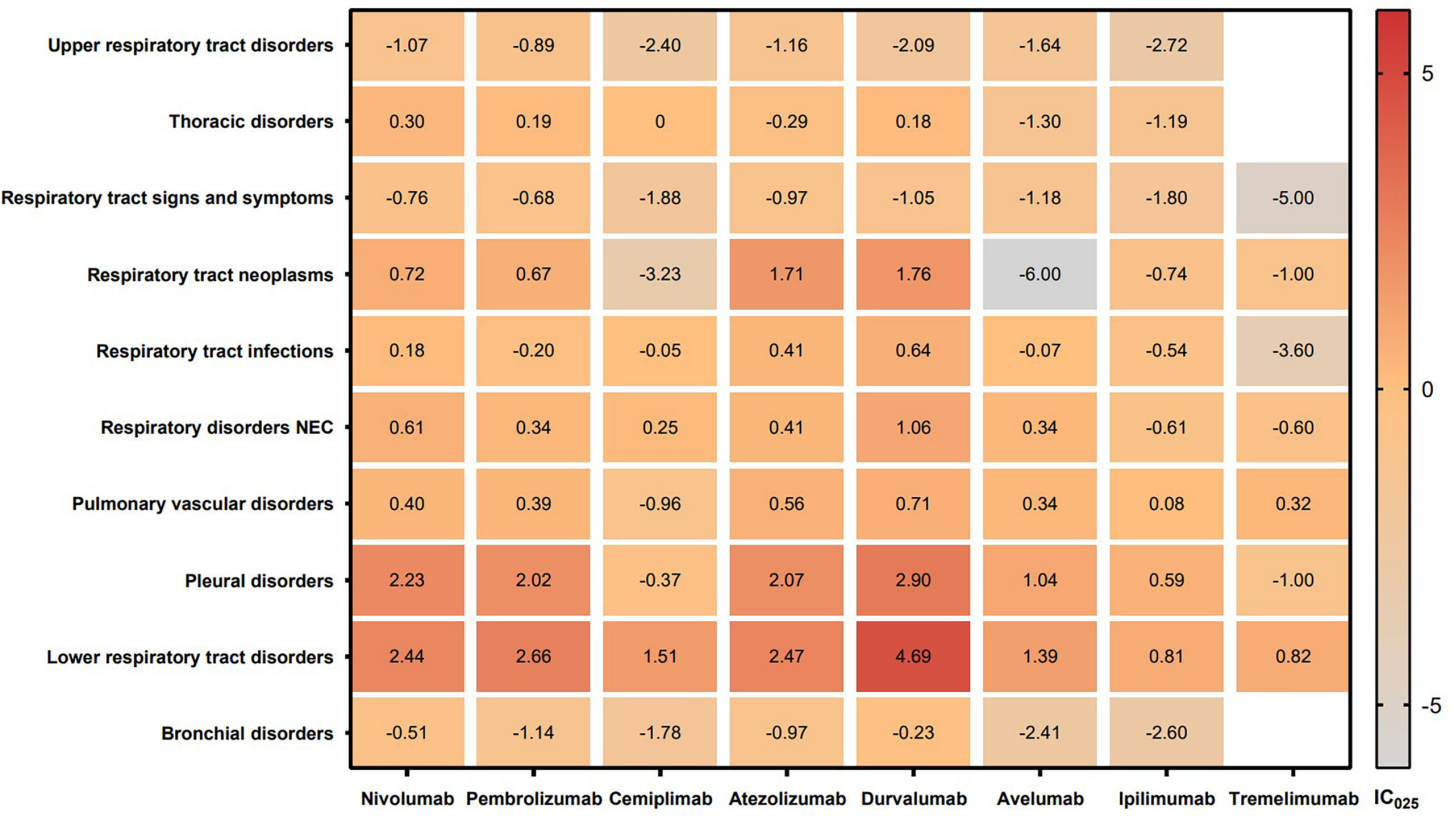
IC_025_ values across class-specific respiratory system toxicities and different ICI subpopulations. ICIs, immune checkpoint inhibitors; IC, information component.

The signal values and the association between class-specific ICIs and respiratory system toxicities are shown in **Table 4**. Regarding different class-specific ICI regimens, anti-PD-1 drugs (nivolumab, pembrolizumab, and cemiplimab) and anti-PD-L1 drugs (atezolizumab, durvalumab, and avelumab) were significantly associated with respiratory system toxicities (**Table 4**). However, anti-CTLA-4 drugs (ipilimumab and tremelimumab) did not demonstrate a significant association with respiratory system toxicities. Upon further analysis, we explored the spectrum of respiratory system toxicities among different ICI subpopulations (**Figure 4**, **Table S5**). Notably, lower respiratory tract disorders had the most significant signals in ICI subpopulations (IC_025_: from 0.82 to 4.69) and then pleural disorders (IC_025_: from 0.59 to 2.90). However, the drugs with the least variety in respiratory system toxicities were cemiplimab and tremelimumab. Lower respiratory tract disorders (IC_025_ = 1.51) and respiratory disorders (NEC) (IC_025_ = 0.25) were the only two AEs with signals detected for cemiplimab. Lower respiratory tract disorders (IC_025_ = 0.82) and pulmonary vascular disorders (IC_025_ = 0.32) were the only two AEs with signals detected for tremelimumab.

**Table 4:** The associations of respiratory system AEs with different ICI regimens.

Drug	N	ROR	ROR_025_	ROR_975_	IC	IC_025_	IC_975_
Nivolumab	13,241	1.47	**1.45**	1.50	0.56	**0.53**	0.58
Pembrolizumab	9,958	1.36	**1.33**	1.38	0.44	**0.41**	0.46
Cemiplimab	215	1.29	**1.12**	1.48	0.37	**0.14**	0.53
Atezolizumab	3,928	1.53	**1.48**	1.59	0.62	**0.56**	0.66
Durvalumab	2,989	3.11	**2.99**	3.25	1.64	**1.58**	1.68
Avelumab	414	1.29	**1.17**	1.43	0.37	**0.21**	0.49
Ipilimumab	1,198	0.74	0.70	0.78	-0.44	-0.54	-0.37
Tremelimumab	25	1.43	0.94	2.16	0.51	-0.16	0.98

AEs, adverse events; ICIs, immune checkpoint inhibitors; N, number of records; IC_025_, the lower limit of a 95% CI for the IC; IC_975_, the upper limit of a 95% CI; ROR_025_, the lower limit of the 95% CI of ROR; ROR_975_, the upper limit of the 95% CI of ROR. IC_025_ >0 and ROR_025_ >1 were deemed a signal (bold mark).

**Figure 5:**
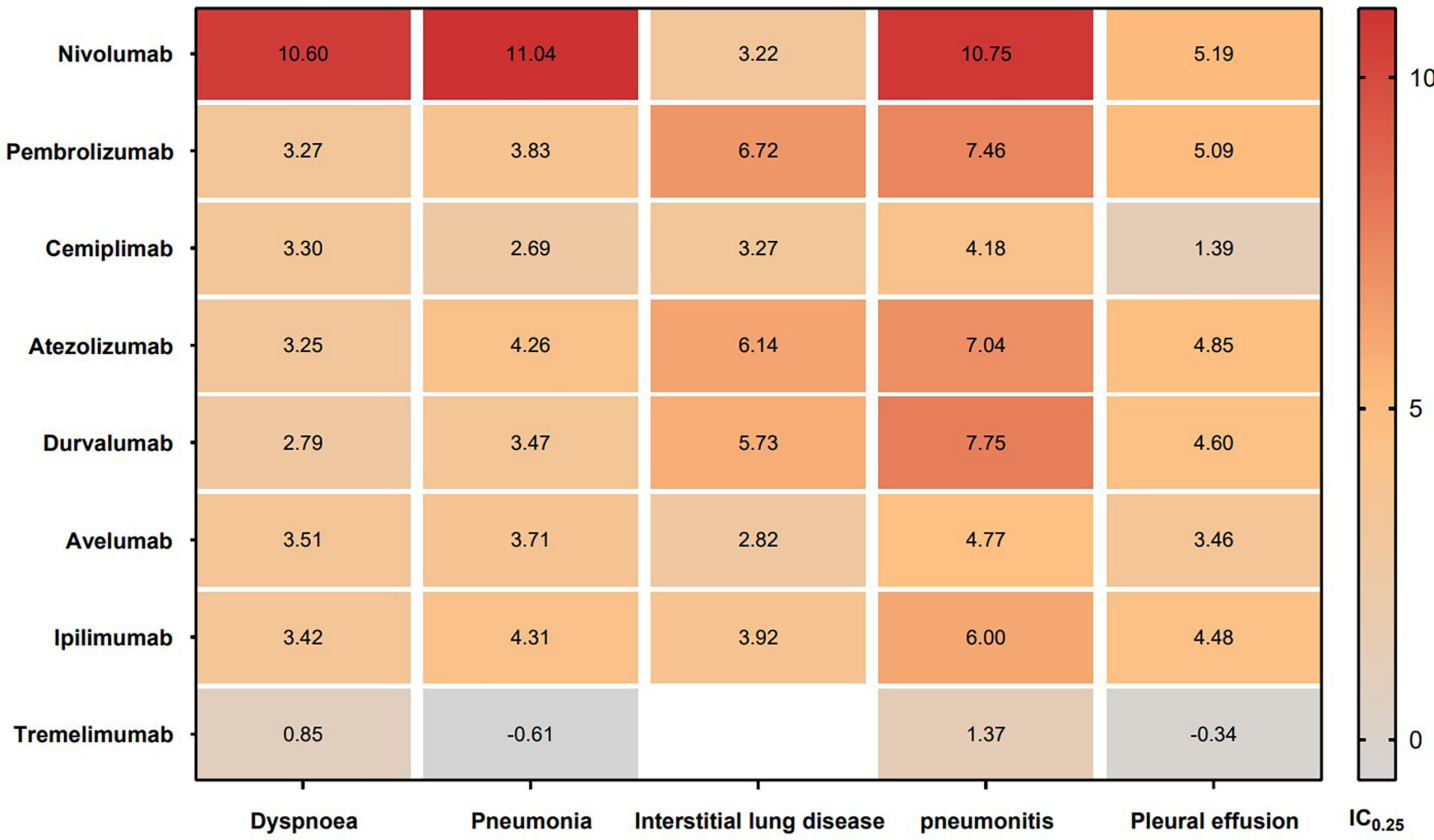
IC_025_ values across class-specific ICIs and the top 5 most frequently reported PTs of respiratory system AEs in the FAERS database. ICIs, immune checkpoint inhibitors; AEs, adverse events; IC, information component.

In addition, the top 5 most frequently reported PTs of respiratory system AEs are shown in **Figure 5**. Dyspnea, interstitial lung disease, and pneumonitis were found to be significantly associated with all eight types of ICIs. Pneumonia and pleural effusion were found to be significantly associated with seven types of ICIs excluding tremelimumab. Nivolumab had most significant signals in dyspnea (IC_025_ = 10.60), pneumonia (IC_025_ = 11.04), and pneumonitis (IC_025_ = 10.75). Pembrolizumab, atezolizumab and durvalumab had relatively high signals in interstitial lung disease (IC_025_: 6.72, 6.14, 5.73) and pneumonitis (IC_025_: 7.46, 7.04, 7.75).

## Discussion

ICIs have suggested remarkable clinical benefits in multiple types of cancer. With the increasing frequency of use, ICIs has contributed to significant survival improvements in cancer patients (3, 4, 11). However, there is growing evidence that the use of ICIs is related to a higher-than-expected rate of respiratory system risks. Since the first clinical trials on ICIs, there have been multiple reports of respiratory system AEs (22–25). However, details for these AEs remain unclear. There is an urgent need to find the respiratory system toxicity profile after ICI administration from real-world evidence. To our knowledge, the FAERS database is a typical spontaneous reporting system (SRS), which includes the most extensive and exhaustive characterization of ICI-associated respiratory system toxicities. We employed the FAERS database to analyze the clinical features, spectrum, onset time, and outcomes of ICI-associated respiratory system toxicities.

First, from January 2014 to September 2021, the reporting rate for ICI-related respiratory system toxicities was approximately 0.12%, which, to some extent, suggested that ICI-related respiratory system toxicities remain rare and that the reporting rate is low. However, most cases were reported in 2017–2021, suggesting the substantially increased use of ICIs in recent years. In **Table 2**, the descriptive analysis was more likely to demonstrate overreported respiratory system AEs in men than in women. After further disproportionality analysis, the results showed that men had a slightly higher reporting frequency than that of women (ROR = 1.74, 95% CI: 1.70–1.78). This result may be due to men being more exposed to cigarette smoke (26, 27). However, this result may also be attributed to a higher cancer incidence in men than that in women and the overrepresentation of men treated with ICIs (28–30), but not explicitly, necessitating further evidence to verify this result. To date, few studies have explored sex differences in ICI-induced AEs and much fewer have analyzed those in respiratory system AEs. Furthermore, an increasing number of studies have reported that the efficacy of immunotherapy varies between male and female patients, with greater efficacy in male patients (28, 29). Therefore, sex should be regarded as an important factor in further studies, especially in respiratory system AEs. In addition, the proportion of respiratory system AEs between patients aged ≥70 years and those aged<70 years was not significantly different (ROR = 0.86, 95% CI: 0.84–0.88). This result is similar to that of Paderi et al. (31).

Second, although several studies have reported that ICI-associated respiratory system toxicities occur early, the time to onset of class-specific events after the administration of ICIs is still unclear (16, 32). This study partially fills this gap and provides more information for future studies. We found that the median onset time of respiratory system AEs after ICI administration was 36 days, and the majority of these events occurred within 3 months. Importantly, the differential spectra of time-to-onset according to different types of respiratory system AEs were analyzed in this study. We found that the median time to onset occurred fairly early for upper respiratory tract disorders (28 days, Q1–Q3: 14–91 days) and was most delayed for pulmonary vascular disorders and thoracic disorders (42 days, Q1–Q3: 14–93 days; 42 days, Q1–Q3: 17–119 days, respectively). It is worthwhile to recognize the difference in time to onset between class-specific respiratory system AEs; furthermore, these results highlight an importance of early and close follow-up after ICI treatment, particularly in the first 3 months, to ensure early intervention in affected populations.

Third, we found that death accounted for 30.64% of all associated ICI-related respiratory system AE records. Considering that the descriptive analysis was more likely to be affected by overreporting, further disproportionality analysis was conducted in this study. The results suggested that ICI-associated respiratory system AEs had a slightly higher reporting frequency than those of other drug-associated respiratory system AEs (ROR = 2.74, 95% CI: 2.68–2.80), implying a significant impact of respiratory system complications on the mortality of patients. The study by Suresh et al. (33) showed that the presence of ICI-related respiratory system toxicities, such as pneumonitis, increases the mortality of patients. Our results, to some extent, agree with this finding. Given the potential mortality of different types of respiratory system AEs, early and intensified monitoring is particularly necessary.

Fourth, we did note that the anti-CTLA-4 drugs had a lower degree of association with respiratory system toxicities than that of anti-PD-1/anti-PD-L1 drugs. This result is similar to those of the studies of Nishino et al. (15) and Naidoo et al. (17). However, the mechanism remains to be further analyzed but may be secondary to the expression of programmed death ligand in pulmonary cells (34). Our study also provides more data on the spectrum of respiratory system AEs induced by different ICI regimens. These results suggested that 7 in 10 class-specific respiratory system AEs (lower respiratory tract disorders, pleural disorders, pulmonary vascular disorders, respiratory disorders NEC, respiratory tract infections, respiratory tract neoplasms, and thoracic disorders) were associated with ICIs. Among these AEs, respiratory disorders NEC, lower respiratory tract disorders, and respiratory tract infections largely comprised the reported problems. Notably, the magnitude of the disproportionality association was the highest for lower respiratory tract disorders. Upon further analysis of PTs exhibited in lower respiratory tract disorders, interstitial lung disease and pneumonitis were found to be significantly associated with all eight types of ICIs, revealing interstitial lung disease and pneumonitis as the primary focus of current immunotherapy studies. Since the first clinical trials on both ICIs, interstitial lung disease and pneumonitis associated with ICI treatment have been reported. According to a meta-analysis, the overall ICI-related interstitial lung disease incidence was 2.7% for all grades and 0.8% for the most severe grades (grade ≥3) (15). The overall incidence of ICI-related interstitial lung disease for all grades was between 1.4% and 5.8% in non-small-cell lung cancer (NSCLC) studies, whereas in renal cell carcinoma studies, it was between 2.7% and 11.8% (15, 35). Another study suggested that the incidence of all grades of immune-related pneumonitis induced by ICIs was 6.2% in NSCLC patients (36). However, the exact mechanism of pneumonitis or interstitial lung disease related to PD-1 blockade is unclear. It has been suggested that T lymphocytes can regulate the function of macrophage and dendritic cell during acute infection (37). Furthermore, PD-1 could induce a negative feedback to weaken the innate immunoinflammatory responses and the damage of tissue elicited by Toll-like receptors and cytokine signaling (37). Moreover, besides pneumonitis and interstitial lung disease, pleural effusion was also a common PT in ICI-related respiratory system AEs.

Several limitations in our study should be acknowledged. First, the FAERS database has limitations itself, with multiple data sources, a nonuniform data format, data duplication, and missing data. Second, the FAERS database does not provide detailed clinical information. Third, a case report could include several drugs and/or several AEs. We took a combination of drug–AE pairs as the basic unit rather than the report, so the results from this pharmacovigilance analysis may be subject to bias. Fourth, we only analyzed ICI monotherapy but failed to consider the combined use of ICIs. Nevertheless, our study quantified the potential risks systematically and scientifically with the large data and described a spectrum of the occurrence of ICI-related respiratory system toxicities, which could provide valuable evidence for further studies and clinical practice in this field.

## Conclusions

This study showed a high reporting frequency of respiratory system toxicities caused by ICIs. Early recognition and management of ICI-related respiratory system AEs are of vital importance in practice. Maximizing the benefit while reducing potential respiratory system toxicities of ICIs should be-come a priority.

## Data availability statement

The original contributions presented in the study are included in the article/**Supplementary Material**. Further inquiries can be directed to the corresponding authors.

## Author contributions

CC performed the data analysis and drafted the manuscript. LD performed the analysis of the data and collaborated in discussing findings. XR and WW performed the analysis of the data. WC and YW conceived and designed the study and revised the manuscript. All authors contributed to the article and approved the submitted version.

## Funding

This study was supported by National Natural Science Foundation of China (Grant No. 82072361) and Beijing Chaoyang District Science and technology Plan CYSF2030.

## Conflict of interest

The authors declare that the research was conducted in the absence of any commercial or financial relationships that could be construed as a potential conflict of interest.

## Publisher’s note

All claims expressed in this article are solely those of the authors and do not necessarily represent those of their affiliated organizations, or those of the publisher, the editors and the reviewers. Any product that may be evaluated in this article, or claim that may be made by its manufacturer, is not guaranteed or endorsed by the publisher.
