# Supplementary material for: Respiratory system toxicity induced by immune checkpoint inhibitors: A real-world study based on the FDA adverse event reporting system database
Source: Front Oncol. 2022 Aug 19;12:941079. doi: 10.3389/fonc.2022.941079 (PMC9437516; doi:10.3389/fonc.2022.941079)
Supplement: Supplementary file 1 [file Table_1.docx]

**Table S1** Included PT below the SOC of respiratory system

| **HLGT** | **HLT** | **PT** |
| --- | --- | --- |
| Upper respiratory tract disorders (excl infections) | Tracheal disorders (excl infections and neoplasms) |  |
|  |  | Tracheo-oesophageal fistula |
|  |  | Tracheal haemorrhage |
|  |  | Tracheal obstruction |
|  |  | Acquired tracheo-oesophageal fistula |
|  |  | Tracheal fistula |
|  |  | Tracheomalacia |
|  |  | Tracheal disorder |
|  |  | Tracheostomy malfunction |
|  |  | Tracheal squamous cell metaplasia |
|  |  | Tracheal radiation injury |
|  |  | Tracheal inflammation |
|  |  | Tracheal deviation |
|  |  | Tracheal mass |
|  |  | Tracheal injury |
|  |  | Tracheal stenosis |
|  | Pharyngeal disorders (excl infections and neoplasms) |  |
|  |  | Pharyngeal inflammation |
|  |  | Pharyngeal haemorrhage |
|  |  | Pharyngeal swelling |
|  |  | Pharyngeal ulceration |
|  |  | Pharyngeal disorder |
|  |  | Pharyngeal erythema |
|  |  | Tonsillar disorder |
|  |  | Tonsillar hypertrophy |
|  |  | Pharyngeal hypoaesthesia |
|  |  | Pharyngeal paraesthesia |
|  |  | Pharyngeal fistula |
|  |  | Dysaesthesia pharynx |
|  |  | Pharyngeal necrosis |
|  |  | Pharyngeal erosion |
|  |  | Oropharyngeal spasm |
|  |  | Pharyngeal lesion |
|  |  | Tonsillolith |
|  |  | Pharyngeal stenosis |
|  |  | Oropharyngeal swelling |
|  |  | Pharyngeal mass |
|  |  | Oropharyngeal dysplasia |
|  |  | Pharyngeal oedema |
|  | Paranasal sinus disorders (excl infections and neoplasms) |  |
|  |  | Sinus congestion |
|  |  | Paranasal sinus hypersecretion |
|  |  | Sinonasal obstruction |
|  |  | Chronic eosinophilic rhinosinusitis |
|  |  | Allergic sinusitis |
|  |  | Paranasal sinus haemorrhage |
|  |  | Paranasal sinus mucosal hypertrophy |
|  |  | Sinus polyp |
|  |  | Sinus disorder |
|  | Nasal disorders NEC |  |
|  |  | Parosmia |
|  |  | Nasal dryness |
|  |  | Anosmia |
|  |  | Nasal polyps |
|  |  | Nasal disorder |
|  |  | Nasal injury |
|  |  | Nasal septum deviation |
|  |  | Nasal pruritus |
|  |  | Nasal ulcer |
|  |  | Nasal septum perforation |
|  |  | Nasal cavity mass |
|  |  | Nasal turbinate hypertrophy |
|  |  | Nasal crusting |
|  |  | Nasal mucosal discolouration |
|  |  | Nasal mucosa atrophy |
|  |  | Nasal septum ulceration |
|  |  | Epistaxis |
|  | Nasal congestion and inflammations |  |
|  |  | Seasonal allergy |
|  |  | Rhinitis allergic |
|  |  | Vasomotor rhinitis |
|  |  | Nasal inflammation |
|  |  | Nasal congestion |
|  | Laryngeal spasm, oedema and obstruction |  |
|  |  | Laryngeal oedema |
|  |  | Stridor |
|  |  | Epiglottic oedema |
|  |  | Laryngeal obstruction |
|  |  | Laryngeal stenosis |
|  |  | Angioedema |
|  | Laryngeal and adjacent sites disorders NEC (excl infections and neoplasms) |  |
|  |  | Paralysis recurrent laryngeal nerve |
|  |  | Vocal cord paresis |
|  |  | Vocal cord disorder |
|  |  | Laryngeal haemorrhage |
|  |  | Laryngeal inflammation |
|  |  | Vocal cord dysfunction |
|  |  | Laryngeal disorder |
|  |  | Vocal cord leukoplakia |
|  |  | Laryngeal dyspnoea |
|  |  | Epiglottic erythema |
|  |  | Vocal cord thickening |
|  |  | Radiation larynx injury |
|  |  | Laryngeal ulceration |
|  |  | Vocal cord paralysis |
| Thoracic disorders (excl lung and pleura) | Thoracic musculoskeletal disorders |  |
|  |  | Respiratory muscle weakness |
|  |  | Post-thoracotomy pain syndrome |
|  |  | Deformity thorax |
|  |  | Musculoskeletal chest pain |
|  | Mediastinal disorders |  |
|  |  | Pneumomediastinum |
|  |  | Hilar lymphadenopathy |
|  |  | Mediastinal disorder |
|  |  | Mediastinum neoplasm |
|  |  | Metastases to the mediastinum |
|  |  | Mediastinitis |
|  |  | Pulmonary hilum mass |
|  |  | Pulmonary hilar enlargement |
|  |  | Mediastinal mass |
|  |  | Thymoma |
|  |  | Malignant neoplasm of thymus |
|  |  | Mediastinal haemorrhage |
|  |  | Mediastinal abscess |
|  |  | Neoplasm of thymus |
|  |  | Thymic cancer metastatic |
|  |  | Mediastinal fibrosis |
|  |  | Mediastinal shift |
|  |  | Staphylococcal mediastinitis |
|  |  | Oesophagomediastinal fistula |
|  |  | Lymphadenopathy mediastinal |
|  | Diaphragmatic disorders |  |
|  |  | Diaphragmatic paralysis |
|  |  | Diaphragm muscle weakness |
|  |  | Diaphragmatic disorder |
|  |  | Diaphragmatic hernia |
|  |  | Acquired diaphragmatic eventration |
|  |  | Diaphragmatic spasm |
|  |  | Hiatus hernia |
| Respiratory tract signs and symptoms | Upper respiratory tract signs and symptoms |  |
|  |  | Oropharyngeal pain |
|  |  | Speech disorder |
|  |  | Rhinorrhoea |
|  |  | Aphonia |
|  |  | Throat irritation |
|  |  | Throat tightness |
|  |  | Choking |
|  |  | Sneezing |
|  |  | Oropharyngeal discomfort |
|  |  | Dry throat |
|  |  | Upper-airway cough syndrome |
|  |  | Upper respiratory tract inflammation |
|  |  | Sinus headache |
|  |  | Choking sensation |
|  |  | Laryngeal discomfort |
|  |  | Increased upper airway secretion |
|  |  | Nasal discomfort |
|  |  | Sinus pain |
|  |  | Paranasal sinus discomfort |
|  |  | Laryngeal pain |
|  |  | Snoring |
|  |  | Oropharyngeal plaque |
|  |  | Nasal obstruction |
|  |  | Catarrh |
|  |  | Oropharyngeal blistering |
|  |  | Rhinalgia |
|  |  | Throat clearing |
|  |  | Upper respiratory tract congestion |
|  |  | Nasopharyngeal reflux |
|  |  | Throat lesion |
|  |  | Yawning |
|  |  | Upper airway obstruction |
|  |  | Increased viscosity of upper respiratory secretion |
|  |  | Dysphonia |
|  | Respiratory signs and symptoms NEC |  |
|  |  | Chest discomfort |
|  |  | Non-cardiac chest pain |
|  |  | Painful respiration |
|  |  | Respiratory symptom |
|  |  | Suffocation feeling |
|  |  | Diaphragmalgia |
|  |  | Hypertrophic osteoarthropathy |
|  |  | Nasal flaring |
|  |  | Clubbing |
|  |  | Chest pain |
|  | Lower respiratory tract signs and symptoms |  |
|  |  | Hiccups |
|  |  | Lung opacity |
|  |  | Pleuritic pain |
|  |  | Pulmonary pain |
|  |  | Rales |
|  |  | Lower respiratory tract congestion |
|  |  | Rhonchi |
|  |  | Increased bronchial secretion |
|  |  | Increased viscosity of bronchial secretion |
|  |  | Lung hyperinflation |
|  |  | Lung hypoinflation |
|  |  | Pulmonary haemorrhage |
| Respiratory tract neoplasms | Upper respiratory tract neoplasms |  |
|  |  | Oropharyngeal squamous cell carcinoma |
|  |  | Laryngeal cancer |
|  |  | Nasal cavity cancer |
|  |  | Pharyngeal cancer |
|  |  | Nasal sinus cancer |
|  |  | Pharyngeal cancer metastatic |
|  |  | Nasopharyngeal cancer |
|  |  | Tracheal cancer |
|  |  | Tracheal papilloma |
|  |  | Nasopharyngeal cancer recurrent |
|  |  | Hypopharyngeal cancer recurrent |
|  |  | Laryngeal cancer stage IV |
|  |  | Metastases to larynx |
|  |  | Benign tracheal neoplasm |
|  |  | Tracheal neoplasm |
|  |  | Nasopharyngeal cancer stage IV |
|  |  | Laryngeal cancer metastatic |
|  |  | Squamous cell carcinoma of pharynx |
|  |  | Metastases to trachea |
|  |  | Nasal neoplasm |
|  |  | Paranasal sinus and nasal cavity malignant neoplasm |
|  |  | Laryngeal neoplasm |
|  |  | Postcricoid cancer |
|  |  | Throat cancer |
|  | Respiratory tract neoplasms NEC |  |
|  |  | Metastases to thorax |
|  | Lower respiratory tract neoplasms |  |
|  |  | Lung neoplasm malignant |
|  |  | Non-small cell lung cancer |
|  |  | Lung adenocarcinoma |
|  |  | Small cell lung cancer |
|  |  | Lung cancer metastatic |
|  |  | Lung neoplasm |
|  |  | Non-small cell lung cancer recurrent |
|  |  | Squamous cell carcinoma of lung |
|  |  | Lung carcinoma cell type unspecified recurrent |
|  |  | Non-small cell lung cancer metastatic |
|  |  | Non-small cell lung cancer stage IV |
|  |  | Bronchial carcinoma |
|  |  | Lung carcinoma cell type unspecified stage IV |
|  |  | Lung adenocarcinoma stage IV |
|  |  | Non-small cell lung cancer stage III |
|  |  | Lung adenocarcinoma recurrent |
|  |  | Lung carcinoma cell type unspecified stage 0 |
|  |  | Metastatic bronchial carcinoma |
|  |  | Lung squamous cell carcinoma metastatic |
|  |  | Bronchial neoplasm |
|  |  | Carcinoid tumour pulmonary |
|  |  | Small cell lung cancer recurrent |
|  |  | Small cell lung cancer extensive stage |
|  |  | Small cell lung cancer metastatic |
|  |  | Lung squamous cell carcinoma stage IV |
|  |  | Lung infiltration malignant |
|  |  | Bronchioloalveolar carcinoma |
|  |  | Non-small cell lung cancer stage IIIA |
|  |  | Adenosquamous cell lung cancer |
|  |  | Large cell lung cancer |
|  |  | Neuroendocrine tumour of the lung metastatic |
|  |  | Sarcomatoid carcinoma of the lung |
|  |  | Lung squamous cell carcinoma stage III |
|  |  | Lung adenocarcinoma stage III |
|  |  | Small cell lung cancer limited stage |
|  |  | Neuroendocrine tumour of the lung |
|  |  | Lung squamous cell carcinoma recurrent |
|  |  | Lung carcinoma cell type unspecified stage III |
|  |  | Metastases to lung |
| Respiratory tract infections | Viral upper respiratory tract infections |  |
|  |  | Viral upper respiratory tract infection |
|  |  | Respiratory syncytial virus infection |
|  |  | H1N1 influenza |
|  |  | Nasal herpes |
|  |  | Herpes pharyngitis |
|  |  | Viral pharyngitis |
|  |  | COVID-19 |
|  |  | COVID-19 pneumonia |
|  |  | Pneumonia viral |
|  |  | Suspected COVID-19 |
|  |  | Pneumonia cytomegaloviral |
|  |  | Pneumonia influenzal |
|  |  | Bronchitis viral |
|  |  | Metapneumovirus infection |
|  |  | Asymptomatic COVID-19 |
|  |  | Pneumonia respiratory syncytial viral |
|  |  | Lower respiratory tract infection viral |
|  |  | Severe acute respiratory syndrome |
|  |  | Pneumonia herpes viral |
|  |  | Influenza |
|  | Upper respiratory tract infections NEC |  |
|  |  | Upper respiratory tract infection |
|  |  | Sinusitis |
|  |  | Pharyngitis |
|  |  | Rhinitis |
|  |  | Laryngitis |
|  |  | Tracheitis |
|  |  | Epiglottitis |
|  |  | Chronic sinusitis |
|  |  | Tonsillitis |
|  |  | Oropharyngeal candidiasis |
|  |  | Acute sinusitis |
|  |  | Pharyngeal abscess |
|  |  | Peritonsillar abscess |
|  |  | Pharyngotonsillitis |
|  |  | Peritonsillitis |
|  |  | Sinobronchitis |
|  |  | Nasopharyngitis |
|  | Respiratory tract infections NEC |  |
|  |  | Coronavirus infection |
|  |  | Respiratory tract infection viral |
|  |  | Parainfluenzae virus infection |
|  |  | Respiratory tract infection bacterial |
|  |  | Mucormycosis |
|  |  | Respiratory moniliasis |
|  |  | Disseminated mucormycosis |
|  |  | Respiratory tract infection |
|  | Lower respiratory tract infections NEC |  |
|  |  | Pneumonia aspiration |
|  |  | Bronchitis |
|  |  | Lower respiratory tract infection |
|  |  | Lung abscess |
|  |  | Pulmonary sepsis |
|  |  | Atypical pneumonia |
|  |  | Pneumonia necrotising |
|  |  | Tracheobronchitis |
|  |  | Pneumonia mycoplasmal |
|  |  | Paracancerous pneumonia |
|  |  | Bronchopulmonary aspergillosis allergic |
|  |  | Post procedural pneumonia |
|  |  | Pneumonia |
|  | Fungal upper respiratory tract infections |  |
|  |  | Oropharyngitis fungal |
|  |  | Pneumocystis jirovecii pneumonia |
|  |  | Bronchopulmonary aspergillosis |
|  |  | Pneumonia fungal |
|  |  | Aspergilloma |
|  |  | Pneumonia cryptococcal |
|  |  | Candida pneumonia |
|  |  | Coccidioidomycosis |
|  |  | Lower respiratory tract infection fungal |
|  |  | Sinusitis fungal |
|  | Bacterial upper respiratory tract infections |  |
|  |  | Tonsillitis bacterial |
|  |  | Upper respiratory tract infection bacterial |
|  |  | Pharyngitis bacterial |
|  |  | Pertussis |
|  |  | Bacterial rhinitis |
|  |  | Pneumonia bacterial |
|  |  | Pulmonary tuberculosis |
|  |  | Pneumonia pneumococcal |
|  |  | Pneumonia klebsiella |
|  |  | Pneumonia staphylococcal |
|  |  | Pneumonia pseudomonal |
|  |  | Pulmonary nocardiosis |
|  |  | Pneumonia streptococcal |
|  |  | Pneumonia legionella |
|  |  | Bronchitis bacterial |
|  |  | Lower respiratory tract infection bacterial |
|  |  | Pneumonia moraxella |
|  |  | Pneumonia escherichia |
|  |  | Pneumonia haemophilus |
|  |  | Pneumonia chlamydial |
|  |  | Atypical mycobacterial pneumonia |
|  |  | Pneumonia serratia |
|  |  | Enterobacter pneumonia |
|  |  | Pharyngitis streptococcal |
| Respiratory disorders NEC | Respiratory tract disorders NEC |  |
|  |  | Pulmonary mass |
|  |  | Respiratory disorder |
|  |  | Aspiration |
|  |  | Respiratory tract congestion |
|  |  | Respiratory tract inflammation |
|  |  | Respiratory tract oedema |
|  |  | Bronchopneumopathy |
|  |  | Respiratory tract irritation |
|  |  | Respiratory tract haemorrhage |
|  |  | Foreign body in throat |
|  |  | Dependence on respirator |
|  |  | Allergic respiratory disease |
|  |  | Thoracic haemorrhage |
|  |  | Respiratory tract procedural complication |
|  |  | Foreign body aspiration |
|  |  | Foreign body in respiratory tract |
|  |  | Lung disorder |
|  | Respiratory failures (excl neonatal) |  |
|  |  | Acute respiratory failure |
|  |  | Cardio-respiratory arrest |
|  |  | Cardiopulmonary failure |
|  |  | Chronic respiratory failure |
|  |  | Respiratory paralysis |
|  |  | Cardio-respiratory distress |
|  |  | Postoperative respiratory failure |
|  |  | Postoperative respiratory distress |
|  |  | Respiratory failure |
|  | Coughing and associated symptoms |  |
|  |  | Haemoptysis |
|  |  | Productive cough |
|  |  | Sputum discoloured |
|  |  | Sputum retention |
|  |  | Sputum increased |
|  |  | Sputum purulent |
|  |  | Post-tussive vomiting |
|  |  | Cough |
|  | Conditions associated with abnormal gas exchange |  |
|  |  | Cyanosis |
|  |  | Asphyxia |
|  |  | Hypercapnia |
|  |  | Respiratory acidosis |
|  |  | Hypercapnic coma |
|  |  | Hypoxic-ischaemic encephalopathy |
|  |  | Anoxia |
|  |  | Respiratory alkalosis |
|  |  | Brain hypoxia |
|  |  | Cyanosis central |
|  |  | Hypobarism |
|  |  | Hyperoxia |
|  |  | Hypoxia |
|  | Breathing abnormalities |  |
|  |  | Respiratory distress |
|  |  | Dyspnoea exertional |
|  |  | Tachypnoea |
|  |  | Respiratory arrest |
|  |  | Hypopnoea |
|  |  | Respiratory depression |
|  |  | Dyspnoea at rest |
|  |  | Respiration abnormal |
|  |  | Apnoea |
|  |  | Hyperventilation |
|  |  | Orthopnoea |
|  |  | Sleep apnoea syndrome |
|  |  | Hypoventilation |
|  |  | Respiratory fatigue |
|  |  | Bradypnoea |
|  |  | Grunting |
|  |  | Pickwickian syndrome |
|  |  | Prolonged expiration |
|  |  | Transfusion-associated dyspnoea |
|  |  | Dyspnoea paroxysmal nocturnal |
|  |  | Cheyne-Stokes respiration |
|  |  | Cardiac asthma |
|  |  | Irregular breathing |
|  |  | Dyspnoea |
| Pulmonary vascular disorders | Vascular pulmonary disorders NEC |  |
|  |  | Hepatopulmonary syndrome |
|  |  | Pulmonary veno-occlusive disease |
|  |  | Pulmonary arteriovenous fistula |
|  |  | Pulmonary artery stenosis |
|  |  | Pulmonary vein occlusion |
|  |  | Pulmonary vascular disorder |
|  |  | Pulmonary artery occlusion |
|  | Pulmonary thrombotic and embolic conditions |  |
|  |  | Pulmonary thrombosis |
|  |  | Pulmonary artery thrombosis |
|  |  | Pulmonary infarction |
|  |  | Pulmonary venous thrombosis |
|  |  | Septic pulmonary embolism |
|  |  | Pulmonary tumour thrombotic microangiopathy |
|  |  | Pulmonary microemboli |
|  |  | Post procedural pulmonary embolism |
|  |  | Pulmonary embolism |
|  | Pulmonary hypertensions |  |
|  |  | Pulmonary arterial hypertension |
|  |  | Cor pulmonale |
|  |  | Cor pulmonale acute |
|  |  | Pulmonary hypertension |
| Pleural disorders | Pneumothorax and pleural effusions NEC |  |
|  |  | Pneumothorax |
|  |  | Haemothorax |
|  |  | Pneumothorax spontaneous |
|  |  | Hydrothorax |
|  |  | Chylothorax |
|  |  | Procedural pneumothorax |
|  |  | Pneumothorax traumatic |
|  |  | Traumatic haemothorax |
|  |  | Paraneoplastic pleural effusion |
|  |  | Eosinophilic pleural effusion |
|  |  | Pleural effusion |
|  | Pleural neoplasms |  |
|  |  | Metastases to pleura |
|  |  | Pleural mesothelioma malignant |
|  |  | Pleural mesothelioma |
|  |  | Malignant neoplasm of pleura |
|  |  | Malignant pleural effusion |
|  | Pleural infections and inflammations |  |
|  |  | Infectious pleural effusion |
|  |  | Pleural infection |
|  |  | Pleuropericarditis |
|  |  | Tuberculous pleurisy |
|  |  | Pyopneumothorax |
|  |  | Pleural infection bacterial |
|  |  | Pleurisy |
|  | Pleural conditions NEC |  |
|  |  | Pleural disorder |
|  |  | Pleural thickening |
|  |  | Pleural fistula |
|  |  | Pleural mass |
|  |  | Oesophagopleural fistula |
|  |  | Pleural calcification |
|  |  | Bronchopleural fistula |
| Lower respiratory tract disorders (excl obstruction and infection) | Pulmonary oedemas |  |
|  |  | Acute respiratory distress syndrome |
|  |  | Acute pulmonary oedema |
|  |  | Pulmonary congestion |
|  |  | Non-cardiogenic pulmonary oedema |
|  |  | Acute lung injury |
|  |  | Pulmonary oedema |
|  | Parenchymal lung disorders NEC |  |
|  |  | Organising pneumonia |
|  |  | Atelectasis |
|  |  | Pulmonary toxicity |
|  |  | Pulmonary fibrosis |
|  |  | Lung infiltration |
|  |  | Pulmonary alveolar haemorrhage |
|  |  | Emphysema |
|  |  | Lung consolidation |
|  |  | Idiopathic pulmonary fibrosis |
|  |  | Traumatic lung injury |
|  |  | Pulmonary cavitation |
|  |  | Engraftment syndrome |
|  |  | Acute interstitial pneumonitis |
|  |  | Lung cyst |
|  |  | Pulmonary necrosis |
|  |  | Restrictive pulmonary disease |
|  |  | Pulmonary fistula |
|  |  | Cystic lung disease |
|  |  | Alveolar proteinosis |
|  |  | Combined pulmonary fibrosis and emphysema |
|  |  | Bronchopulmonary disease |
|  |  | Antisynthetase syndrome |
|  |  | Alveolar lung disease |
|  |  | Complications of transplanted lung |
|  |  | Idiopathic interstitial pneumonia |
|  |  | Middle lobe syndrome |
|  |  | Pulmonary interstitial emphysema syndrome |
|  |  | Lung perforation |
|  |  | Interstitial lung disease |
|  | Occupational parenchymal lung disorders |  |
|  |  | Silicosis |
|  |  | Pneumoconiosis |
|  |  | Pneumonitis chemical |
|  | Lower respiratory tract radiation disorders |  |
|  |  | Pulmonary radiation injury |
|  |  | Radiation pneumonitis |
|  | Lower respiratory tract inflammatory and immunologic conditions |  |
|  |  | Immune-mediated lung disease |
|  |  | Eosinophilic pneumonia |
|  |  | Pulmonary sarcoidosis |
|  |  | Alveolitis |
|  |  | Hypersensitivity pneumonitis |
|  |  | Diffuse alveolar damage |
|  |  | Pulmonary granuloma |
|  |  | Autoimmune lung disease |
|  |  | Eosinophilic granulomatosis with polyangiitis |
|  |  | Granulomatosis with polyangiitis |
|  |  | Graft versus host disease in lung |
|  |  | Pulmonary vasculitis |
|  |  | Eosinophilic pneumonia acute |
|  |  | Granulomatous pneumonitis |
|  |  | Diffuse panbronchiolitis |
|  |  | Pneumonia lipoid |
|  |  | Pulmonary eosinophilia |
|  |  | Eosinophilia myalgia syndrome |
|  |  | Lower respiratory tract inflammation |
|  |  | Pneumonitis |
| Bronchial disorders (excl neoplasms) | Bronchospasm and obstruction |  |
|  |  | Wheezing |
|  |  | Asthma |
|  |  | Bronchospasm |
|  |  | Obstructive airways disorder |
|  |  | Bronchial obstruction |
|  |  | Bronchostenosis |
|  |  | Bronchitis chronic |
|  |  | Infective exacerbation of chronic obstructive airways disease |
|  |  | Obliterative bronchiolitis |
|  |  | Bronchial hyperreactivity |
|  |  | Bronchial oedema |
|  |  | Asthmatic crisis |
|  |  | Reactive airways dysfunction syndrome |
|  |  | Cystic fibrosis |
|  |  | Chronic obstructive pulmonary disease |
|  | Bronchial conditions NEC |  |
|  |  | Bronchiolitis |
|  |  | Bronchial fistula |
|  |  | Oesophagobronchial fistula |
|  |  | Bronchial disorder |
|  |  | Bronchial secretion retention |
|  |  | Bronchial haemorrhage |
|  |  | Bronchial wall thickening |
|  |  | Eosinophilic bronchitis |
|  |  | Bronchial injury |
|  |  | Bronchial ulceration |
|  |  | Bronchiectasis |

**Table S2** Summary of ICIs drug name in FAERS database

| Target | Standard name | Other names |
| --- | --- | --- |
| PD-1 | nivolumab | OPDIVO, MDX-1106, ONO4538, BMS-936558 |
|  | pembrolizumab | KEYTRUDA, MK-3475 |
|  | cemiplimab | LIBTAYO, SAR439684, REGN2810 |
| PD-L1 | atezolizumab | TECENTRIQ, MPDL3280A |
|  | durvalumab | IMFINZI, MEDI4736 |
|  | avelumab | BAVENCIO, MSB0010718C |
| CTLA-4 | ipilimumab  tremelimumab | YERVOY, BMS-734016, MDX-010  TICILIMUMAB |

**Table S3** The time to onset with ICIs-related class-specific respiratory system AEs

| **Respiratory system events** | **Time to onset (days)** | | | | | | |
| --- | --- | --- | --- | --- | --- | --- | --- |
|  | 0-30d | 31-90d | 91-180d | 181-270d | 271-360d | >360d | Data available |
| Bronchial disorders, n | 229 | 87 | 55 | 23 | 13 | 29 | 436 |
| Lower respiratory tract disorders, n | 1935 | 1141 | 533 | 225 | 90 | 190 | 4114 |
| Pleural disorders, n | 678 | 333 | 177 | 63 | 34 | 29 | 1314 |
| Pulmonary vascular disorders, n | 319 | 216 | 92 | 28 | 31 | 34 | 720 |
| Respiratory disorders NEC, n | 3061 | 1298 | 718 | 229 | 138 | 223 | 5667 |
| Respiratory tract infections, n | 1825 | 1015 | 542 | 221 | 119 | 191 | 3913 |
| Respiratory tract neoplasms, n | 125 | 53 | 39 | 9 | 8 | 7 | 241 |
| Respiratory tract signs and symptoms, n | 632 | 224 | 95 | 55 | 22 | 37 | 1065 |
| Thoracic disorders, n | 64 | 28 | 26 | 5 | 4 | 5 | 132 |
| Upper respiratory tract disorders, n | 236 | 72 | 44 | 22 | 8 | 15 | 397 |
| Proportion, (%) | 50.58% | 24.82% | 12.90% | 4.89% | 2.59% | 4.22% | 100.00% |

**Table S4** The median time to onset with ICIs-related class-specific respiratory system AEs

| **Respiratory system events** | **Time to onset (days)** | |
| --- | --- | --- |
|  | Median | Quartile1-3 |
| Total | 36 | 14-98 |
| Bronchial disorders | 34 | 14-115 |
| Lower respiratory tract disorders | 38 | 14-97 |
| Pleural disorders | 34 | 12-90 |
| Pulmonary vascular disorders | 42 | 14-93 |
| Respiratory disorders NEC | 32 | 12-95 |
| Respiratory tract infections | 41 | 14-107 |
| Respiratory tract neoplasms | 36 | 11-102 |
| Respiratory tract signs and symptoms | 30 | 12-85 |
| Thoracic disorders | 42 | 17-119 |
| Upper respiratory tract disorders | 28 | 14-91 |

| **Respiratory system events** | **Nivolumab** | | | | | **Pembrolizumab** | | | | **Cemiplimab** | | | | **Atezolizumab** | | | | **Durvalumab** | | | | **Avelumab** | | | | **Ipilimumab** | | | | **Tremelimumab** | | | | |
| --- | --- | --- | --- | --- | --- | --- | --- | --- | --- | --- | --- | --- | --- | --- | --- | --- | --- | --- | --- | --- | --- | --- | --- | --- | --- | --- | --- | --- | --- | --- | --- | --- | --- | --- |
|  | **N** | | **IC** | **IC_025_** | **IC_975_** | **N** | **IC** | **IC_025_** | **IC_975_** | **N** | **IC** | **IC_025_** | **IC_975_** | **N** | **IC** | **IC_025_** | **IC_975_** | **N** | **IC** | **IC_025_** | **IC_975_** | **N** | **IC** | **IC_025_** | **IC_975_** | **N** | **IC** | **IC_025_** | **IC_975_** | **N** | **IC** | **IC_025_** | **IC_975_** |  |
| **Upper respiratory tract disorders** | 358 | -0.90 | | -1.07 | -0.77 | 332 | -0.71 | -0.89 | -0.58 | 6 | -0.99 | -2.40 | -0.07 | 106 | -0.84 | -1.16 | -0.61 | 26 | -1.44 | -2.09 | -0.97 | 14 | -0.74 | -1.64 | -0.12 | 28 | -2.09 | -2.72 | -1.64 | 0 | 0 | 0 | 0 |  |
| **Thoracic disorders** | 118 | 0.60 | | 0.30 | 0.82 | 92 | 0.54 | 0.19 | 0.79 | 0 | 0.00 | 0.00 | 0.00 | 28 | 0.34 | -0.29 | 0.78 | 17 | 1.00 | 0.18 | 1.56 | 4 | 0.47 | -1.30 | 1.55 | 12 | -0.22 | -1.19 | 0.45 | 0 | 0 | 0 | 0 |  |
| **Respiratory tract signs and symptoms** | 965 | -0.65 | | -0.76 | -0.57 | 835 | -0.57 | -0.68 | -0.48 | 14 | -0.98 | -1.88 | -0.36 | 253 | -0.77 | -0.97 | -0.62 | 98 | -0.72 | -1.05 | -0.48 | 35 | -0.61 | -1.18 | -0.21 | 99 | -1.46 | -1.80 | -1.22 | 1 | -1.2 | -5 | 0.5 |  |
| **Respiratory tract neoplasms** | 340 | 0.90 | | 0.72 | 1.03 | 272 | 0.87 | 0.67 | 1.01 | 2 | -0.63 | -3.23 | 0.76 | 201 | 1.94 | 1.71 | 2.11 | 86 | 2.12 | 1.76 | 2.37 | 1 | -2.22 | -6.00 | -0.53 | 30 | -0.13 | -0.74 | 0.30 | 2 | 1.55 | -1 | 2.94 |  |
| **Respiratory tract infections** | 2722 | 0.25 | | 0.18 | 0.29 | 1729 | -0.12 | -0.20 | -0.06 | 56 | 0.39 | -0.05 | 0.71 | 934 | 0.52 | 0.41 | 0.59 | 428 | 0.80 | 0.64 | 0.92 | 98 | 0.26 | -0.07 | 0.50 | 324 | -0.36 | -0.54 | -0.22 | 2 | -1 | -3.6 | 0.41 |  |
| **Respiratory disorders NEC** | 4349 | 0.66 | | 0.61 | 0.69 | 2970 | 0.40 | 0.34 | 0.44 | 79 | 0.62 | 0.25 | 0.89 | 1121 | 0.51 | 0.41 | 0.59 | 670 | 1.19 | 1.06 | 1.28 | 150 | 0.61 | 0.34 | 0.81 | 367 | -0.44 | -0.61 | -0.32 | 8 | 0.54 | -0.6 | 1.35 |  |
| **Pulmonary vascular disorders** | 430 | 0.56 | | 0.40 | 0.68 | 352 | 0.56 | 0.39 | 0.69 | 7 | 0.35 | -0.96 | 1.20 | 148 | 0.83 | 0.56 | 1.03 | 68 | 1.12 | 0.71 | 1.41 | 22 | 1.05 | 0.34 | 1.55 | 73 | 0.47 | 0.08 | 0.75 | 4 | 2.08 | 0.32 | 3.16 |  |
| **Pleural disorders** | 939 | 2.34 | | 2.23 | 2.42 | 668 | 2.14 | 2.02 | 2.24 | 7 | 0.93 | -0.37 | 1.79 | 256 | 2.27 | 2.07 | 2.42 | 178 | 3.14 | 2.90 | 3.32 | 23 | 1.73 | 1.04 | 2.22 | 67 | 0.99 | 0.59 | 1.29 | 2 | 1.54 | -1 | 2.94 |  |
| **Lower respiratory tract disorders** | 2599 | 2.50 | | 2.44 | 2.55 | 2477 | 2.73 | 2.66 | 2.78 | 37 | 2.06 | 1.51 | 2.45 | 783 | 2.58 | 2.47 | 2.67 | 1353 | 4.78 | 4.69 | 4.84 | 59 | 1.83 | 1.39 | 2.14 | 173 | 1.06 | 0.81 | 1.24 | 6 | 2.23 | 0.82 | 3.14 |  |
| **Bronchial disorders** | 421 | -0.35 | | -0.51 | -0.23 | 231 | -0.92 | -1.14 | -0.76 | 7 | -0.48 | -1.78 | 0.38 | 98 | -0.64 | -0.97 | -0.39 | 65 | 0.18 | -0.23 | 0.48 | 8 | -1.20 | -2.41 | -0.40 | 25 | -1.93 | -2.60 | -1.46 | 0 | 0 | 0 | 0 |  |

**Table S5** The associations of respiratory system AEs with different ICIs subpopulations

**Table S6** The frequency of reported PT in Lower respiratory tract disorders

| **PT** | **Frequency** |
| --- | --- |
| Pneumonitis | 3366 |
| Interstitial lung disease | 2378 |
| Radiation pneumonitis | 814 |
| Pulmonary oedema | 333 |
| Organising pneumonia | 246 |
| Immune-mediated lung disease | 173 |
| Acute respiratory distress syndrome | 173 |
| Atelectasis | 150 |
| Pulmonary toxicity | 124 |
| Pulmonary fibrosis | 121 |
| Lung infiltration | 106 |
| Pulmonary alveolar haemorrhage | 72 |
| Emphysema | 65 |
| Acute pulmonary oedema | 47 |
| Eosinophilic pneumonia | 45 |
| Pulmonary congestion | 44 |
| Pulmonary sarcoidosis | 42 |
| Alveolitis | 35 |
| Hypersensitivity pneumonitis | 35 |
| Lung consolidation | 33 |
| Idiopathic pulmonary fibrosis | 27 |
| Diffuse alveolar damage | 23 |
| Traumatic lung injury | 20 |
| Pulmonary granuloma | 17 |
| Autoimmune lung disease | 16 |
| Eosinophilic granulomatosis with polyangiitis | 15 |
| Pneumonitis chemical | 13 |
| Pulmonary radiation injury | 12 |
| Non-cardiogenic pulmonary oedema | 12 |
| Granulomatosis with polyangiitis | 12 |
| Pulmonary cavitation | 11 |
| Engraftment syndrome | 11 |
| Acute interstitial pneumonitis | 10 |
| Graft versus host disease in lung | 9 |
| Lung cyst | 9 |
| Pulmonary necrosis | 9 |
| Acute lung injury | 8 |
| Restrictive pulmonary disease | 7 |
| Pulmonary vasculitis | 6 |
| Pulmonary fistula | 5 |
| Cystic lung disease | 5 |
| Eosinophilic pneumonia acute | 5 |
| Alveolar proteinosis | 4 |
| Combined pulmonary fibrosis and emphysema | 4 |
| Granulomatous pneumonitis | 4 |
| Bronchopulmonary disease | 3 |
| Antisynthetase syndrome | 3 |
| Diffuse panbronchiolitis | 2 |
| Alveolar lung disease | 2 |
| Complications of transplanted lung | 2 |
| Idiopathic interstitial pneumonia | 2 |
| Silicosis | 1 |
| Middle lobe syndrome | 1 |
| Pneumonia lipoid | 1 |
| Pulmonary interstitial emphysema syndrome | 1 |
| Lung perforation | 1 |
| Pulmonary eosinophilia | 1 |
| Eosinophilia myalgia syndrome | 1 |
| Pneumoconiosis | 1 |
| Lower respiratory tract inflammation | 1 |
